# Extracellular vesicles in TDP-43 proteinopathies: pathogenesis and biomarker potential

**DOI:** 10.1186/s13024-025-00859-4

**Published:** 2025-06-10

**Authors:** Elizabeth R. Dellar, Lara Nikel, Stephanie Fowler, Björn F. Vahsen, Ruxandra Dafinca, Emily Feneberg, Kevin Talbot, Martin R. Turner, Alexander G. Thompson

**Affiliations:** 1 Nuffield Department of Clinical Neurosciences, University of Oxford, Level 6, West Wing John Radcliffe Hospital, OX3 9DU Oxford, UK; 2 Kavli Institute for Nanoscience Discovery, University of Oxford, Dorothy Crowfoot Hodgkin Building, Oxford, UK; 3 Oxford-GSK Institute of Molecular and Computational Medicine, University of Oxford, Oxford, UK; 4 Institute of Neurology, University College London, London, UK; 5 Department of Neurology, Technical University of Munich School of Medicine and Health, Munich, Germany

**Keywords:** Extracellular vesicle, Exosome, Neurodegeneration, Amyotrophic lateral sclerosis, Frontotemporal dementia, TDP-43.

## Abstract

Extracellular vesicles (EVs) are membrane-enclosed nanoparticles released by most cell types, and from multiple sub-cellular compartments. They carry a range of cargo biomolecules, including protein and RNA that reflect the type and status of their cell of origin. EVs are associated with the 43 kDa trans-active response DNA binding protein (TDP-43), aggregates of which are a key pathological feature across the spectrum of amyotrophic lateral sclerosis (ALS), frontotemporal dementia (FTD), and other TDP-43 proteinopathies. The secretion of TDP-43 via EVs may have beneficial effects through the cellular clearance of aggregated protein, or detrimental effects via the intercellular spread of templated TDP-43 aggregation. This review examines evidence for the association of TDP-43 with EVs in *post mortem* tissue, in vitro models and human biofluids, and discusses evidence for EV TDP-43 involvement in pathogenesis via clearance or ‘seeding’ processes. We further discuss the potential for use of EVs in biomarker development, through analysis of TDP-43 or alternative protein and RNA cargoes.

**Graphical Abstract:**

## Abstract (de)

**Graphical Abstract:**

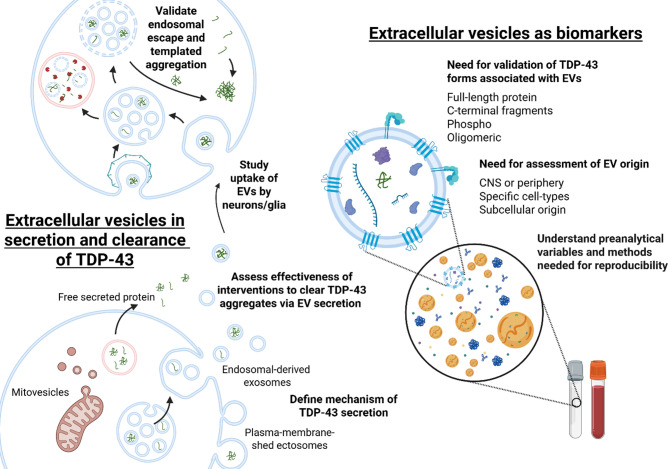

## Introduction

Trans-active response DNA binding protein 43 kDa (TDP-43) is an RNA-binding protein primarily localised to the nucleus with functions in regulation of splicing, translation and stability of RNA, and cellular stress responses [1, 2]. The presence of cytoplasmic aggregates comprising full-length phosphorylated and ubiquitinated TDP-43 and 15–35 kDa C-terminal fragments within neurons and glia of the nervous system is a feature of several overlapping neurodegenerative syndromes, collectively termed TDP-43 proteinopathies. TDP-43 aggregates within the brain and/or spinal cord are the core neuropathological hallmark of over 95% of cases of amyotrophic lateral sclerosis (ALS) and around 50% of cases of frontotemporal dementia (FTD). TDP-43-related FTD cases are clinically indistinguishable from FTD cases associated with the aggregation of microtubule-associated protein tau (MAPT) [3, 4]. TDP-43 aggregation within the limbic system is also the main feature of amnestic dementia cases (limbic predominant age-related TDP-43 encephalopathy - LATE), and up to 65% of Alzheimer’s Disease (AD) cases [5–7]. LATE and AD are also clinically indistinguishable, and LATE is thought to account for up to 20% of clinical AD diagnoses. TDP-43 pathology is also recognised in several rare diseases including Perry disease: a progressive Parkinsonian syndrome with neuropsychiatric features and hypoventilation due to dominantly inherited rare variants in the dynactin-1 gene; and multisystem proteinopathy – a dominantly inherited disorder manifesting with Paget’s disease of bone, inclusion body myositis, ALS or FTD, and occurring due to variants in valosin-containing protein (*VCP*) and sequestosome-1 (*SQSTM1*) genes [8, 9]. Most TDP-43 proteinopathies do not represent defined genetic syndromes and develop in the absence of an identifiable monogenic cause. A subset of TDP-43 proteinopathies can present in the context of rare genetic variants, such as hexanucleotide repeat expansion (HRE) in an intronic region of the *C9orf72* gene (*C9orf72* HRE) which accounts for around 10% of cases of ALS and FTD, and variants in the granulin precursor (*GRN*), which accounts for 5–10% of FTD caes [10–14].

Alongside the neuropathological evidence for the central role of TDP-43 in these diseases, variants in *TARDBP* (encoding TDP-43) are found in a small percentage of cases of ALS and FTD [15]. Most disease-causing variants in *TARDBP* are found in the aggregate-forming glycine-rich C-terminal domain, and some of these have also been demonstrated to increase the aggregation propensity of TDP-43 in vitro [16]. This implicates TDP-43 aggregation as one of a number of possible mechanisms for direct TDP-43 involvement in ALS pathogenesis. Nuclear clearance and loss-of-function of TDP-43 may also be key features of the disease process [17]. Recent research has highlighted the effect of a reduction in TDP-43-mediated splice repression activity due to nuclear depletion, which results in the inclusion of intronic regions to mature RNAs (cryptic exons) such as *STMN2* and *UNC13A* that are either subjected to nonsense-mediated decay or translated to form truncated or cryptic peptides [18].

Extracellular vesicles (EVs) are lipid membrane-bound nanoparticles that are released by cells into the extracellular environment, including by cells in the brain and spinal cord [19]. EVs were first described as a mechanism for maintenance of cellular homeostasis through the removal of protein from the cell, a function which also expands to removal of lipid, DNA and RNA [20, 21]. This function is especially pertinent to many neurodegenerative diseases, including prion diseases, AD and Parkinson’s disease (PD). Here, protein aggregation is an overarching defining feature, as EVs are suggested as a key mechanism by which prion, tau, beta-amyloid (Aβ) and alpha-synuclein, as well as TDP-43, may be secreted into the extracellular environment [19]. Increasing EV secretion pathways has therefore gained traction as a possible therapeutic strategy by which TDP-43 aggregates can be cleared from cells [22]. In parallel, EVs have been proposed as a vehicle by which protein aggregates might be transmitted between cells, facilitating templated protein aggregation [23]. Some evidence exists suggesting that this may also be the case for TDP-43, thereby contributing to progression of disease in TDP-43 proteinopathies. Whether acting as a vehicle for waste clearance or templated protein aggregation, the presence of TDP-43 in biofluid-derived EVs alone has great relevance as a potential biomarker in TDP-43 proteinopathies [24, 25].

Aside from a possible role as a carrier of TDP-43, changes to extracellular vesicle secretion and composition in TDP-43 proteinopathies have been observed, which may have mechanistic relevance in understanding of the cellular pathogenic mechanisms and provide biomarker potential beyond TDP-43 protein itself. This review will consider the evidence for a pathogenic role of EVs and their potential biomarker utility in TDP-43 proteinopathies, with an evaluation of how the methodologies applied for their extraction and analysis may influence conclusions drawn from these investigations.

## Extracellular vesicles and the importance of enrichment methodology

**Fig. 1:**
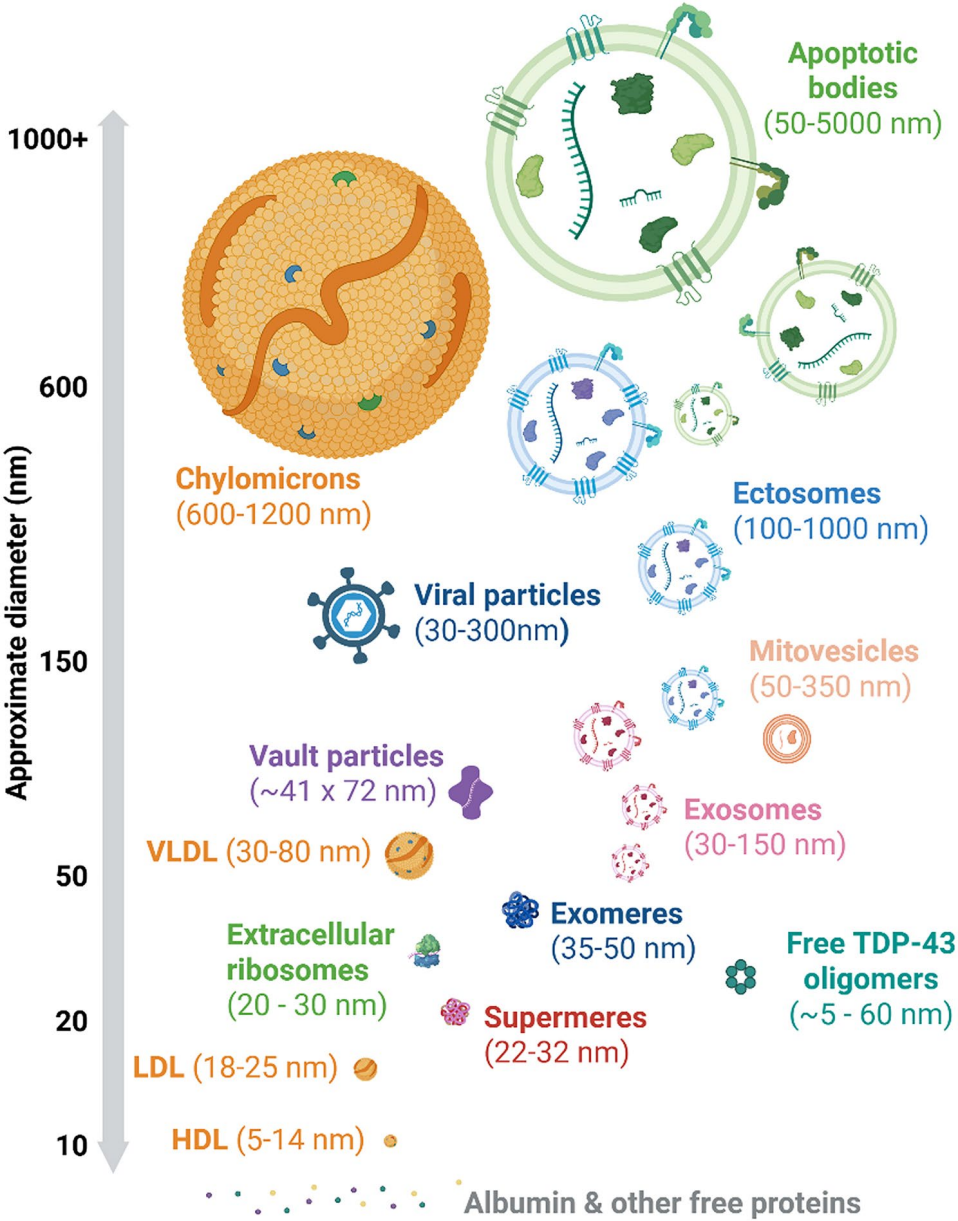
Extracellular particle types that can carry protein or RNA in biofluids. Schematic showing an overview of extracellular vesicles (Exosomes, ectosomes, mitovesicles, apoptotic bodies) and non-vesicular particles including High-density lipoprotein (HDL), Low-density lipoprotein (LDL), very low-density lipoprotein (VLDL), chylomicrons, exomeres, supermeres, extracellular ribosomes, vault particles and protein oligomers. Sizes shown are approximate, demonstrating overlapping characteristics between different particle types and the challenge of specific separation. Figure created with BioRender.com

Multiple studies have identified the presence of TDP-43 in EV preparations enriched by a range of methods from different sources including post-mortem tissue, plasma, CSF, and cell culture media (Table 1). However, EVs are one of many different extracellular particle types which frequently co-isolate in common enrichment strategies (Fig. 1). The ability to determine the precise nature of the association of TDP-43 with EVs is strongly influenced by the methodology employed. This is exemplified by more recent studies on other aggregate-forming proteins (tau, alpha-synuclein and Aβ), which have indicated that only a small fraction (0–3%) of the total extracellular forms of these proteins are directly EV-associated, with the majority present in free extracellular form [26–28]. One commonly used EV purification method (5 studies in Table 1) makes use of polymer-based precipitation reagents. This is cheap and simple to carry out, but is highly non-specific, as it cannot distinguish between EV and other non-vesicular extracellular proteins that co-precipitate [29]. Similarly, whilst increasing purity relative to precipitation, differential ultracentrifugation (9 studies in Table 1) separates EVs based on density, but is also prone to co-isolation of significant quantities of free protein, which might include small species of TDP-43 [29]. Spherical oligomeric species of TDP-43 have been reported to exist, ranging approximately 5 nm diameter to 60 nm, thus overlapping greatly with the expected diameter of small EVs [30, 31]. Sucrose or iodixanol density gradient ultracentrifugation can increase purity, but at the cost of lower yield, which limits its application to biofluids such as cerebrospinal fluid (CSF) where sample volumes are more limited [29, 32]. Size exclusion chromatography (SEC; two studies in Table 1) exhibits good separation from free protein, but in biofluids poorly separates from low and very low-density lipoproteins (LDLs and VLDLs) of similar size [32, 33]. Methods such as cation exchange chromatography and nickel bead isolation (one study in Table 1) can also be used to separate EVs from lipoproteins by exploiting surface charge differences (EVs more negatively charged than lipoproteins) to increase purity, especially when used in combination with other methods [34–36].

**Table 1:** Studies reporting presence of TDP-43 in extracellular vesicles

Reference	EV source and purification		Samples tested		TDP-43 species detected	Change in TDP-43		Antibodies
**CSF**								
[146]	10,000 x*g* UC for large EV 100,000 x*g* UC for small EV		ALS (*n* = 9) FTLD (*n* = 4) HC (*n* = 8)		WB: 45 kDa in small and large EV (*n* = 1) Mass spectrometry unknown	No difference in TDP-43/FLOT1 in disease		Proteintech 10782-2-AP (N-terminal)
[25]	200,000 x*g* UC for small EV		ALS-FTD (n unclear) HC (n unclear)		WB: 43,35 and 25 kDa	Increase in 43, 35 kDa 25 kDa present only in ALS-FTD		Proteintech rabbit polyclonal
[145]	SEC (EV Second L70, GL Sciences)		Sporadic ALS (*n* = 20) Non-neurodegenerative control (*n* = 10)		Unknown (mass spectrometry, peptide data not available)	No significant difference		N/A
[24]	10,000 x*g* UC for large EV SEC (qEVoriginal, Izon) for small EVs Additional L1CAM immunocapture (Clone 5G3)		*DESCRIBE sub-cohort 2* ALS (*n* = 30)		SIMOA: unknown species	CSF EV TDP-43 correlated with plasma EV TDP-43 in small and larger EV		SIMOA targeting amino acids 203–209 and C-terminal region.
**Plasma**								
[77]	20,000 x*g* UC for large EV 100,000 x*g* UC for small EV		Sporadic ALS (*n* = 30) Healthy control (*n* = 30)		WB: 45 kDa doublet and 20 kDa	Increase in 45 kDa doublet in large EV only		Proteintech mouse monoclonal
[34]	Nickel bead (charge-based) total EV		HC (*n* = 1) ALS (*n* = 3) Disease control (*n* = 2)		WB: 45 kDa doublet	N/A		Cosmo Bio Co. CAC-TIP-PTD-M01 (Ser409/410)
[73]	20,000 x*g* UC for large EV 100,000 x*g* UC for small EV		Sporadic FTLD (*n* = 3) HC (*n* = 3)		WB: 43 and 35 kDa	Increase in 35 kDa TDP-43/annexin A1. No difference at 43 kDa.		Proteintech 12892-1-AP (C-terminal)
[46]	Exoquick (polymer precipitation) followed by immunocapture with SLC1A3 (Clone (ACSA-1), L1CAM (Clone 5G3) or TMEM119 (BioLegend, 853302)		LATE NC – TDP-43 (*n* = 42) LATE NC + TDP-43 (*n* = 22)		Positivity by in-house and Cusabio ELISA	L1CAM EV: No change SLC1A3 EV: Increase TMEM119: increase by in-house, decrease by Cusabio		In-house ELISA: C-terminal (a.a. 261–393) Cusabio ELISA (full-length)
[24]	10,000 x*g* UC for large EV SEC (qEVoriginal, Izon) for small EVs Additional L1CAM immunocapture (Clone 5G3)		*DESCRIBE sub-cohort 2* (HC *n* = 56, ALS *n* = 165, bvFTD *n* = 179, PSP *n* = 163) *Sant Paul cohort* (HC *n* = 50, ALS *n* = 65, ALS-FTD *n* = 58, bvFTD *n* = 50, PSP *n* = 41)		SIMOA: unknown species WB: ~28 kDa in small and large EV	Increased levels in ALS and some TDP-43 positive bvFTD (both small and large EV)		Abcam Ab305694 (N-terminal) SIMOA targeting amino acids 203–209 and C-terminal region.
[47]	Exoquick (polymer precipitation followed by immunocapture with L1CAM (Clone 5G3)		AD (*n* = 24) HC (*n* = 15)		Positivity by Signalway Antibody ELISA	Increased levels in AD		Signalway Antibody ELISA: unknown reactivity.
**Serum**								
[145]	SEC (EV Second L70, GL Sciences)		Sporadic ALS (*n* = 20) Non-neurodegenerative control (*n* = 10)		Unknown (mass spectrometry proteomics, peptide data not available)	No significant difference		N/A
**Brain tissue**								
[22]	Human temporal cortex 20,000 x*g* UC for large EV 100,000 x*g* UC followed by sucrose gradient at 200,000 x*g* for small EV		ALS (*n* = 3) Neurological control (*n* = 3)		WB: 15, 28 and 45 kDa (C-terminal) Extensive banding (N-terminal)	2x increase in total TDP-43/FLOT1 5x increase in 28 kDa TDP-43/FLOT1 15 kDa only in ALS		Proteintech 10782-2-AP (N-terminal) Proteintech 12892-1-AP (C-terminal)
[68]	Human motor cortex Sucrose gradient at 200,000 x*g*		ALS (*n* = 3) Neurological control (*n* = 3)		WB; 28 kDa	Increase in 28 kDa		Proteintech 10782-2-AP (N-terminal)
**Cell models**								
[71]	Human SH-SY5Y; transiently transfected with HA-TDP-43. ExoQuick-TC (polymer precipitation)		*n* = 1		WB: 43 and 35 kDa	N/A		Proteintech monoclonal
[69]	HEK293; transiently transfected myc-TDP-43. 150,000 x*g* UC for small EV		*n* = 1		WB: 45 kDa doublet (myc-tagged and endogenous) Both lumen and membrane-associated by immuno-TEM	N/A		Proteintech 10782-2-AP (N-terminal)
[22]	Endogenous mouse Neuro2a; Neuro2a transiently transfected with V5-tagged WT, A315T, G348C or CTF hTDP-43; endogenous mouse cortical neurons. 20,000 x*g* UC for large EV 100,000 x*g* UC for small EV (sucrose gradient at 200,000 x*g* for validation).		*n* = 1 per construct		WB: Endogenous Neuro2a - absent Transfected Neuro2a − 45 kDa, (25 kDa for transfected CTF) in both small and large EV. Cortical neuron- 45 kDa	N/A		Antibody against V5 tag for transfected cells. Proteintech 10782-2-AP (N-terminal for mouse cortical neurons)
[73]	Endogenous mouse NSC-34 20,000 x*g* UC for large EVs 100,000 x*g* UC for small EVs		*n* = 3		43, 35 and 28 kDa by WB, 35 kDa most prominent (both small and large EV)	N/A		Proteintech 12892-1-AP (C-terminal)
[226]	Endogenous human lymphoblastoid cell lines. Invitrogen total exosome isolation reagent (polymer precipitation)		AD (*n* = 3) Healthy control (*n* = 1)		15, 28 and 35 kDa by WB	All species higher in AD		Proteintech 67345-1-Ig (C-terminal)
[75]	Endogenous human lymphoblastoid cell lines. Invitrogen total exosome isolation reagent (polymer precipitation)		FTLD with *GRN* (*n* = 9) Healthy control (*n* = 3)		25 and 50 kDa (phospho) 43 kDa	Increase in phospho CTF in *GRN* heterozygotes. No difference in full length		Proteintech 10782-2-AP (N-terminal) Millipore, MABN14 (Ser409/410)
[70]	Human lymphocyte derived iPSC differentiated to motor neurons. PEG 6000 (polymer precipitation)		*C9orf72* ALS (*n* = 3 lines, 3 replicates) Healthy control (*n* = 3 lines, 3 replicates)		45 and 55 kDa by WB (total) 55 kDa with phospho antibody	No difference ALS and healthy control Increase in phospho TDP-43 with apilimod		Proteintech 10782-2-AP (N-terminal) Proteintech 22309-1-AP (Ser409/410)
[76]	HeLa transiently transfected with GFP-TDP-43 (full-length, N or C-terminal only) Endogenous human SH-SY5Y; Endogenous mouse NSC-34 20,000 x*g* UC for large EV 110,000 x*g* UC for small EV		*n* = 3 for all cell types		WB: HeLa – 35 and 45 kDa. SH-SY5Y- primarily 35 kDa NSC-34–35 and 45 kDa. Present in both small and large EV.	Increased levels with bafilomycin or concanamycin A (*GRN*-dependent)		Proteintech 60019-2-AP (HeLa, SH-SY5Y) Proteintech 12892-1-AP (C-terminal; NSC-34) Proteintech 10782-2-AP (N-terminal; HeLa)
[74]	Endogenous human lymphoblastoid cell lines. Invitrogen total exosome isolation reagent (polymer precipitation)		Sporadic ALS (*n* = 4) Healthy control (*n* = 2)		28 kDa by WB with C-terminal antibody, 35 kDa and 45 kDa with N-terminal antibody	No clear differences		Proteintech 10782-2-AP (N-terminal) Proteintech 67345-1-Ig (C-terminal)
[72]	Endogenous mouse NSC-34 (but transiently transfected with A4V, G37R, G85R, G93A or WT mSOD1) 20,000 x*g* UC for large EV 100,000 x*g* UC for small EV		*n* = 1 per construct		28, 35 and 45 kDa by WB in small EVs, 45 kDa only in large EVs	N/A		Proteintech 12892-1-AP (C-terminal)

HC, healthy control; ALS, amyotrophic lateral sclerosis; FTLD, frontotemporal Lobar degeneration; PSP, progressive supranuclear palsy; AD, alzheimer’s disease; EV, extracellular vesicle; CSF, cerebrospinal fluid; UC, ultracentrifugation; SEC, size-exclusion chromatography; WB, Western blot; TEM, transmission electron microscopy; SLC1A3, solute carrier family 1 member 3, also known as GLAST, glutamate/aspartate transporter 1; L1CAM, L1 cell adhesion molecule; TMEM119, transmembrane protein 119

Crude separation by physical characteristics of size and density hides the diversity of subcellular origins of EVs: in addition to well-described plasma membrane derived (“microvesicles” or “ectosomes”) and late endosomal (“exosomes”)-origin EVs, EVs derived from mitochondria (“mitovesicles”), recycling endosome and amphisome compartments have also been described [29, 37–39]. The subcellular source of EV-associated TDP-43 is likely to be more pathophysiologically relevant than just size, so defining the source would be a great aid in biomarker development and pathological understanding. Emerging affinity-based methods can be implemented to enable separation of more defined populations of EVs based on their biochemical composition.

EVs are believed to differ in lipid composition as compared to the cell plasma membrane, with enrichment in cholesterol, sphingomyelin, glycosphingolipids and phosphatidylserine and depletion of phosphatidylcholine [40]. Several purification methods which exploit this feature have been developed, including Qiagen ExoEasy kit (proprietary target), lysine peptides targeting phospholipids (e.g. ExoIntact), and phosphatidylserine-specific targeting using the protein T-cell immunoglobulin and mucin domain containing 4 (TIMD-4) [41, 42]. Whilst these methods appear to efficiently capture some EVs, relatively poor knowledge of EV lipid composition to date means that it is unclear how these relate to EVs purified by other existing techniques. Membrane externalisation of phosphatidylserine is a key marker of apoptosis and of platelet activation, so is likely to be more highly enriched on apoptotic bodies and may be biased towards specific cell origins [43].

Protein markers of EVs are better defined than lipids. Tetraspanin membrane proteins such as CD81, CD9 and CD63 are amongst the most highly enriched proteins in EVs and have been used effectively for immunoaffinity capture in cell culture studies and in biofluids [29, 44]. Recent evidence suggests that CD81 and CD9 are heavily enriched on the plasma membrane compared to endosomal enrichment of CD63, so may offer some discrimination of origin [44, 45]. Other studies have attempted to use immunocapture for cell-type specific proteins, to enable separation of EVs by cellular origin, including in TDP-43 proteinopathies (three studies in Table 1) [24, 46, 47]. The cell adhesion molecule L1CAM, for example, has been employed to enrich for neuronal-derived EVs but has been highly controversial [48]. Alternative neuronal EV targets such as Neural cell adhesion molecule 1 (NCAM1), adenosine triphosphatase Na+/K + transporting subunit alpha 3 (ATP1A3), neurexin 3 (NRXN3), neuroligin 3 (NLGN3), microtubule associated protein 1B (MAP1B), glutamate ionotropic receptor AMPA type subunit 2 (GRIA2) and Growth-Associated Protein 43 (GAP-43) have also been proposed but have not yet been more widely tested [49–54]. Furthermore, no current data exist on the enrichment of these proteins on EVs from different neuronal sub-types, such as motor neurons and sensory neurons, which would be valuable for studying the specific cell vulnerabilities observed in different diseases. Other studies have attempted to enrich for microglial EVs with Transmembrane Protein 119 (TMEM119) or Integrin Subunit Alpha M (ITGAM or CD11b), astrocytic EVs with Solute Carrier Family 1 Member 3 (SLC1A3, GLAST or EAAT1), or oligodendrocyte EVs with Myelin oligodendrocyte glycoprotein (MOG) [55–59]. Whilst immunoprecipitation with antibodies targeting a variety of antigens has been studied extensively for the purpose of isolating EVs differing by tissue or cell type of origin, the use of immunoprecipitation methods for separation of EVs by subcellular origin has been little explored but may offer a means to define the secretory pathway of EV-associated TDP-43.

## TDP-43 forms detected in association with extracellular vesicles

Analysis of brain and spinal cord tissue from ALS and FTD cases characteristically show that TDP-43 aggregates are found in the form of a high-molecular weight smear (from 55 kDa up to at least 200 kDa), and truncated C-terminal fragments (CTFs) that migrate as a 25 kDa band in immunoblots and typically exhibit extensive phosphorylation and ubiquitination [15]. Antibodies against phosphorylated forms of TDP-43, particularly pS409/410, produced the most robust staining specific to the cytoplasm of both neurons and glia in the brain of ALS and FTD-TDP-43 cases and in the spinal cord in ALS [60]. These antibodies showed bands distinct to disease cases over controls in immunoblots of sarkosyl-insoluble, urea-soluble fractions from tissue [60]. There may also be some differences in the forms of TDP-43 between cell types, brain and spinal cord regions, and in different diseases; for example, CTFs are more frequent in the brain whilst full-length protein is more abundant in the spinal cord [61–64]. Furthermore, some differences in the size of CTFs have been documented in FTD sub-types, alongside structural differences in TDP-43 aggregates characterised by cryo-electron microscopy [62, 65–67]. Establishing the forms of TDP-43 present in EVs enriched from tissue and biofluids is important to understanding their contribution to disease progression and biomarker potential in different TDP-43 proteinopathies.

In tissue-derived EVs (temporal cortex or motor cortex) from people with sporadic ALS and healthy controls (purified by density gradient ultracentrifugation), both full-length and C-terminal fragments (CTFs) were detected (see Table 1) [22, 68]. Increases in the 28 kDa and 15 kDa CTFs, but not the full-length protein, were characteristic of ALS whilst FTD tissue EVs were not studied [22, 68]. Given that differences in CTF length may be associated with different FTD sub-types, a comparison of TDP-43 forms in EVs from these sub-types, and LATE cases, would be informative. EVs from the spinal cord have not yet been studied yet would be of interest given the reported predominance of full-length TDP-43 in this tissue [4, 61]. Importantly, no reactivity was seen in ALS EVs against a phospho-S409/410 antibody by Iguchi et al., despite confirmed reactivity in sarkosyl-insoluble total tissue in 50% of the cases tested [22].

In vitro studies have examined EV-associated TDP-43 expression in a range of human lymphoblastoid cell lines, mouse NSC-34 (motor-neuron-like hybridoma), human SH-SY5Y (neuroblastoma) and mouse N2a (neuroblastoma), as well as human cancer cell lines HEK293 and HeLa with overexpression of TDP-43 by transient transfection (10 studies in Table 1). Full-length TDP-43 has been detected in EVs in some cases [22, 69–72] whilst others have detected primarily short forms at ~ 25 kDa or 35 kDa [73–76]. In HEK293 overexpression models Feiler et al. used immuno-electron microscopy to demonstrate the presence of full-length TDP-43 both within the EV lumen and in association with the limiting membrane [69]. Few studies have looked at primary cell lines or neuronally differentiated patient-derived induced pluripotent stem cells, which will be necessary to understand the physiological relevance of these findings in models not driven by overexpression of TDP-43 [22,70].

Within the study of neurodegenerative disease, so-called “exosomes” have attracted the most interest, but there are currently no validated methods which can distinguish EVs by subcellular origins, with recent guidelines supporting the use of operational terms based on physical characteristics used for enrichment [29]. TDP-43 and other aggregate-forming proteins can be found at similar or higher abundances in EVs pelleted at both low (“large EVs”) and high (“small EVs”) centrifugation speeds across both cell culture and plasma studies [22, 24, 27, 73, 77]. In mouse NSC-34 cells overexpressing SOD1, only full-length TDP-43 was found in large EV preparations, whilst truncated forms were restricted to small EV preparations [72]. Conversely Casarotto et al. found that 35 kDa TDP-43 was the most abundant species in both small and large EVs in the same cell type [73]. In a mouse N2a cell line model, overexpression of the 25 kDa CTF demonstrated its accumulation in small, rather than large, EV preparations [22]. It is important to consider that different fibrillar, oligomeric or monomeric forms of non-vesicular TDP-43 will co-purify with EVs at different speeds, e.g. 10–20,000 x*g* used to pellet large EVs may pellet larger non-vesicular aggregates, whilst higher 100,000 x*g* speeds used for enrichment of small EVs would be more likely to pellet smaller free protein forms.

Overexpression models offer the possibility to study how TDP-43 may be incorporated into EVs. In human SH-SY5Y cells, overexpressed constructs containing the N-terminus of TDP-43 were the most abundant within EV preparations, and in cellular imaging showed a high level of colocalization with CD63 in multivesicular bodies [78]. In HeLa overexpression models, the C-terminus of TDP-43 was shown to be required for secretion of TDP-43 in response to bafilomycin treatment, with imaging showing an increase in localisation to the plasma membrane compared to full-length TDP-43 [76]. These studies highlight that the subcellular localisation of TDP-43 may be a strong determinant of its incorporation into EVs, with the possibility that different TDP-43 species may be enriched in different EV subpopulations. Pinpointing the exact location and species of TDP-43 associated with EVs is critical for: (a) developing reliable biomarkers to detect and differentiate different diseases, (b) understanding how EVs contribute to disease progression and (c) exploring EVs as potential targets for therapies that could block this spread. Testing of a range of EV markers proteins in preparations enriched by different methods will help to establish whether specific EV sub-populations may be the source of TDP-43 and avoid contradictory results, along with an assessment of relative abundance of co-isolated components, such as soluble albumin [29]. In addition, inclusion of TDP-43 controls such recombinant protein, cellular protein and known cross-reactive molecules such as immunoglobulins will aid confidence in identity of detected species.

## Cellular clearance of TDP-43

Whilst there is evidence to suggest that TDP-43 may be secreted via EVs there is limited understanding of the physiological or pathophysiological role of this process, despite the centrality of this question to understanding the identification of tractable therapeutic targets and in biomarker development. Inhibition of EV secretion using chemical inhibition of neutral sphingomyelinase 2 (nSMase2), or by siRNA-mediated knockdown of RAB27A, resulted in an increase in insoluble and phosphorylated TDP-43 aggregates in N2a cells [22]. The same study demonstrated that this inhibition resulted in deficits in both motor and memory tasks in a mouse model of TDP-43^A315T^ that were not seen in wild-type mice and indicate that inhibiting EV-mediated clearance is detrimental. Furthermore, treatment with bafilomycin, a lysosomal acidification inhibitor, or apilimod (an inhibitor of PIKFYVE kinase which generates phosphoinositide PI(3,5)P_2_, a key regulator of endomembrane homeostasis), both stimulate increased EV secretion of aggregate-forming proteins including TDP-43, huntingtin and alpha-synuclein [70, 73, 76, 79, 80]. Apilimod was additionally shown to reduce levels of intracellular insoluble phosphorylated TDP-43 and dipeptide repeat proteins (DPRs) in cellular, invertebrate and murine models of *C9orf72* ALS [70]. These findings were accompanied by enhanced survival, again suggesting a beneficial effect of EV clearance. The target of apilimod, PIKFYVE kinase, is known to catalyse the reverse reaction of the phosphatase FIG4, for which rare loss-of-function *FIG4* variants have been identified in 2–3% of ALS cases in European populations [81]. This pathway may be important in the pathogenic process of ALS, and as a potential preventative target in TDP-43 proteinopathies. Conversely, other studies have demonstrated that apilimod increases secretion of autophagy receptors (such as Sequestosome-1 (SQSTM1) and Optineurin (OPTN)), and autophagosomal markers (such as LC3-II), but not canonical protein markers of EVs (such as ALIX, Caveolin-1, CD63, CD81, CD9, and flotillins), indicating that further validation is required before concluding that the protective effect of apilimod is mediated by EVs [39, 70, 73, 76, 82]. Protease protection assays also indicate that autophagy receptors SQSTM1 and OPTN in EV preparations can be non-vesicular, raising the question of whether this is also the case for TDP-43 and other aggregation-prone proteins [82]. Therefore, whilst an increase in TDP-43 secretion may appear to provide protective effects, more studies are needed to define the mechanism of secretion, and to understand the long-term effects of extracellular TDP-43 on different cell types within the brain, and in the context of pathology.

## Extracellular vesicles as a possible mediator of templated aggregation of TDP-43

Clinical observations in ALS support the view that there is non-random progression of weakness from a focal site to contiguous anatomical regions, alongside characteristic spatiotemporal patterns of atrophy seen in imaging studies [83–86]. This apparent spread of pathology might occur either between cells in close proximity, or via synaptically connected functional or anatomical networks, resulting from loss of electrophysiological stability, shared molecular vulnerability, or transmission of some mediators of toxicity [84, 85, 87]. In both ALS and FTD, analysis of *post mortem* tissue and simulation (using a computational random walker spread model) provide some support for the spread of phosphorylated TDP-43 pathology in disease progression [88–90]. This idea draws close parallels with other neurodegenerative diseases in which it is proposed that templated protein aggregation, whereby a misfolded protein acts as a seed to induce conformational change in normally folded protein via direct contact, mediates disease progression [91–93].

### Uptake of TDP-43 containing EVs

Intercellular spread of TDP-43, in either EV-associated or free forms, would require it to be taken up by surrounding cells. Possible mechanisms for direct transfer of TDP-43 include trans-synaptic transfer, cell-contact-dependent formation of tunnelling nanotubes (TNTs), and heparin sulphate proteoglycan-mediated uptake of fibrillar aggregates, as well as an EV-mediated mechanism [94–102].

The majority of EV uptake is believed to occur via endocytic pathways including clathrin-mediated, caveolin-dependent and receptor-mediated endocytosis, macropinocytosis, and phagocytosis, although at a very low overall rate in cell line model systems such as HeLa [103, 104]. In different cell types the rate of uptake may differ, presumably reflecting relative usage of these different pathways. For example, neurons were found to take up EVs at a low rate compared to glia, whilst astrocytes internalised a greater number of large EVs (or degraded small EVs faster), and microglia internalised both equally [27]. Other factors may also influence the internalisation of EVs. EVs derived from AD brain tissue showed higher uptake by primary neurons, as did EVs derived from irradiated cells in glioblastoma and other cancer cell lines, potentially relating to differences in surface composition [105–107]. IL-1β-stimulated astrocytes were found to secrete EVs enriched in integrins, which exhibited higher uptake by neurons [108]. Similarly, EVs from cells stressed with hydrogen peroxide showed increased levels of annexin A2, which, when targeted to the EV surface, promoted increased uptake of EVs by non-stressed retinal pigment epithelium cells [109]. This may reflect a shift in subcellular source of the EVs, as integrins are highly abundant on the cell plasma membrane. In addition to the effect of irradiation on EV source cells, evidence suggests that irradiation of recipient cells can increase EV uptake, again by altering the protein composition of the cell surface membrane, including of integrin beta 1 [106,110,111]. TDP-43 secretion in the context of inflammatory stimulation have not yet been studied, but these existing studies indicate that other changes in EV or recipient cell protein composition occurring in TDP-43 proteinopathies, perhaps as a result of changes to proteostasis and autophagy, could significantly modulate the effect of EV-associated TDP-43 on a variety of different cell types in the brain and spinal cord.

The EV-associated form of TDP-43, despite its relative low abundance, was more efficiently internalised by HEK293 cells than the free form, similar to observations made for alpha synuclein and tau [27, 28, 69, 107, 112]. However, whilst intercellular transfer of both full-length and truncated forms of tagged protein was seen between co-cultured differentiated SH-SY5Y cells overexpressing TDP-43, it was not seen when adding exogenously generated EVs, or in a transwell system (which separates cells but allows free movement of cell medium constituents), thus leading the authors to conclude a cell-contact dependent mechanism was responsible rather than EVs [78]. There is also evidence that TNTs play a cellular stress-dependent role in TDP-43 transfer in U251 glioblastoma cells [25] and in lymphocytes [74], so differentiating which mechanism(s) are involved in transfer, especially in vivo, is a key question. Evidence suggests that TDP-43 constructs containing the N-terminal region were the most readily transferred between SH-SY5Y cells, so understanding the transfer of different TDP-43 species will also be required [78].

### Seeding capacity

**Fig. 2:**
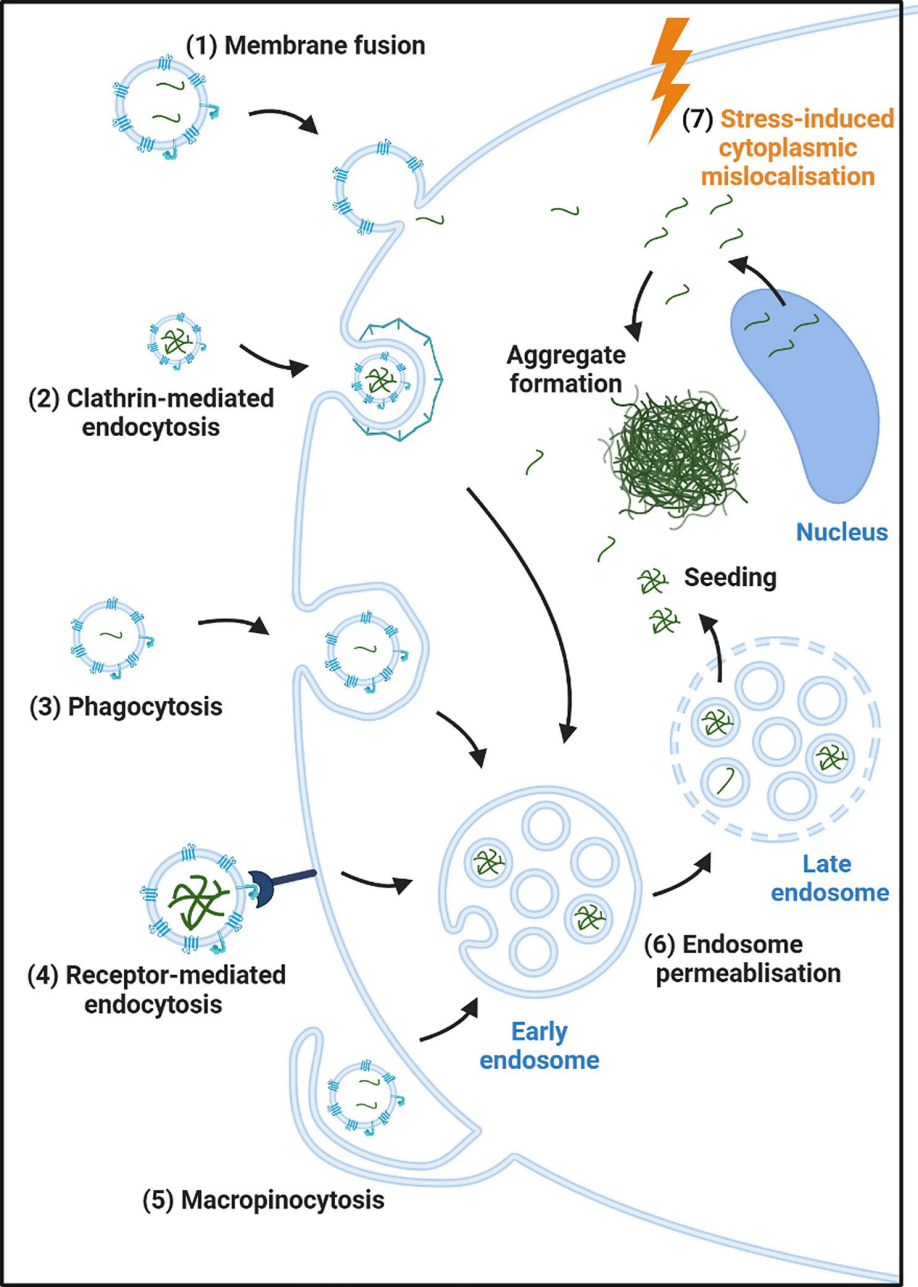
Potential mechanisms of EV-mediated TDP-43 aggregate formation. EVs can be taken up from the extracellular environment by direct plasma membrane fusion (1) or by a variety of different endocytic processes; clathrin-mediated endocytosis (2), phagocytosis (3), receptor-mediated endocytosis (4), or macropinocytosis (5) resulting in localisation of their TDP-43 cargo to the early endosome. For a seeded aggregation process to occur, TDP-43 must escape the endosome to reach the cytoplasm, possibly via a process of endosomal permeabilization (6). Alternatively, EVs might induce cellular stress signalling (7), resulting in nuclear to cytoplasmic mislocalisation of TDP-43, and a subsequent increase in aggregate formation. Different cell types such as neurons and glia may use different mechanisms of uptake which may influence the fate of the internalised TDP-43. Figure created with BioRender.com

For templated protein aggregation to occur, EV-associated TDP-43 would need to reach the cytoplasm to initiate the seeding process **(**Fig. 2**)**. Since endocytosis is believed to be the major route for internalisation of EVs, release of cargo to the cytoplasm would require endosomal escape, which occurs at a low rate in cell models of EV cargo transfer [104, 113–115]. The fate of EV-associated TDP-43 is relatively unknown; however, Polanco et al. identified that the presence of EVs triggered endosomal permeabilisation in around 5% of HEK293T-derived tau biosensor cells, which enabled the release of tau to the cytosol and subsequent templating of cellular tau [116]. Different cell types or sub-types may also show selective vulnerability to this seeding, with in vivo data in a study of tau showing high vulnerability of GABAergic interneurons [107]. Furthermore, in the context of lysosomal dysfunction endosomal escape of EV cargoes appears to increase [104, 113, 115, 117]. The existence of causative variants of ALS and FTD in many genes involved in lysosomal function, including *SQSTM1*,* UBQLN2*,* OPTN*,* VCP*,* TBK1*,* GRN*,* TMEM106B* and *CHMP2B* highlight the potential importance of the process to pathology [118]. Therefore, health of the recipient cells may be the strongest determinant of the seeding capacity of EV-associated proteins.

An additional aspect to consider is whether EV-mediated aggregation of TDP-43 seen in cells is a result of direct templated aggregation, or an indirect result of disruptions to cellular homeostasis **(**Fig. 2**)**. Scant evidence exists to support a direct seeding process for EV-delivered TDP-43; Feiler et al. demonstrated in HEK-293 cells that YFP-tagged TDP-43 was found in the same cytoplasmic granules as endogenous TDP-43 when transferred via conditioned media, but did not demonstrate this colocalisation for the EV-specific fraction [69]. Treatments causing cellular stress (for example TNF-alpha in motor neuron-like NSC-34, sorbitol in HEK-293 cells, and sodium arsenite in iPSC motor neurons), may also be sufficient to induce TDP-43 cytoplasmic mislocalisation and aggregate formation [69, 119–121]. EVs, regardless of any TDP-43 cargo, may also cause a variety of deleterious effects on cells including increased production of reactive oxygen species and DNA damage [122, 123]. Therefore, it is essential to determine whether EVs might be inducing TDP-43 mislocalisation via an indirect effect on cellular stress signalling, rather than a templated aggregation process.

### Involvement of glia

Alongside the possibility of neuron-to-neuron transfer of EVs, there may be a strong influence from glial cells on the fate of EVs in the nervous system. Glial cells, such as microglia and astrocytes, have been shown to be involved in the pathophysiology of TDP-43 proteinopathies, exerting both neuroprotective and neurotoxic effects [124, 125]. One proposed function for EVs is to facilitate the clearance of excess or damaging material, allowing overloaded cells to offload these contents for degradation by other cells in the vicinity [126]. Multiple studies indicate that microglia take up EVs through phagocytosis or macropinocytosis and degrade their contents [127–129]. However, in ALS and FTD, genetic variants in *C9orf72*,* GRN*,* PFN1 and VCP* have been shown to cause reduced endocytic/phagocytic capacity and/or impaired lysosomal acidification in microglia [119, 130–134]. These deficits may compromise the ability of microglia to efficiently clear EV-associated TDP-43, potentially influencing its seeding and spread.

Glial cells are increasingly recognised as active participants in the spread of aggregation-prone proteins in various neurodegenerative diseases, which may involve the release and uptake of EVs. In AD and PD models, microglia have been shown to take up aggregates of tau [135] and α-synuclein [136] and subsequently release them via EVs. Inhibition of microglial EV secretion via knockdown or chemical inhibition of nSMase2 (in the context of inflammatory stimulation with LPS) reduced both secreted tau and levels of tau in neurons incubated with the microglial EVs [135]. Spreading of protein aggregates may be triggered when microglia become overloaded; Brelstaff et al. demonstrated that after phagocytosis of tau-aggregated containing neurons, microglia became hypoactive and rereleased aggregated tau [137]. In astrocyte models, overexpressed SOD1 was found to be associated with EVs which could then be transferred to motor neurons [138]. Whilst direct evidence for a similar mechanism in TDP-43 proteinopathies is limited, in plasma-derived EVs from LATE participants, Winston et al. find higher levels of TDP-43 in EV prepared using SLC1A3 (astrocytic-enriched) and TMEM119 (microglial-enriched) immunocapture, hinting at a possible role for glial-mediated EV transfer of TDP-43 that requires further exploration [46].

Neuroinflammation may further facilitate the mislocalisation, aggregation, and uptake of aggregate-forming proteins. Emerging evidence indicates that EVs from various sources may be immunogenic (due to other protein or RNA cargoes) and can elicit a pro-inflammatory response in microglia and astrocytes, which might be sufficient to induce TDP-43 mislocalisation in these glial cells [27, 139–141]. In ALS, neuroinflammation is strongly associated with disease progression and survival, with evidence suggesting that *C9orf72* HRE microglia exhibit ‘latent neurotoxicity’ that becomes apparent only in response to an additional stimulus [130, 133, 142–144]. In primary neurons incubated with microglial EVs, Guo et al. found that the additional of proinflammatory cytokines, such as TNF-α, IL-1β and IL-6, further increased the burden of insoluble α-synuclein [136]. These data indicate that there may be a complex interplay between the roles of EVs and soluble factors in influencing TDP-43 aggregation in recipient cells.

## Extracellular vesicle TDP-43 as a biomarker

There are currently no specific biomarkers that can be used as a diagnostic biomarker in ALS and FTD. Since the presence of TDP-43 pathology is almost universal in ALS after death, there is a heavy focus on whether it can be reliably detected in biofluids for use a diagnostic and prognostic biomarker. However, detection of relevant TDP-43 is challenging due to its ubiquitous presence in all tissues, not just in the CNS. Cytoplasmic mislocalisation of TDP-43 is widely regarded as a key pathological event in ALS and FTD, so its extracellular release from cells, whether in EV-associated or non-vesicular forms, may act as a direct readout of intracellular pathology. There is therefore great interest in the potential use of EV-associated TDP-43 in biofluids as both a diagnostic and prognostic biomarker in TDP-43 proteinopathies.

In CSF, only four studies have detected TDP-43 in EVs, with 25–28 kDa, 35 kDa and full-length species all reported (see Table 1) [24, 25, 145, 146]. Using non-targeted mass spectrometry for identification, TDP-43 was identified in 8/43 samples from sporadic ALS, and in 0/10 healthy controls in one study, but this was not consistent in longitudinal samples [145]. However again, as for tissue EVs, no studies have confirmed the presence of phosphorylated TDP-43 in CSF EVs.

In blood, TDP-43 was identified in serum-derived EVs in a small proportion of ALS patients but, as for CSF, this was highly inconsistent in longitudinal samples [145]. Using targeted methods in plasma, full-length protein has been detected [24, 34, 46, 77] with an additional 35 kDa species present in FTD cases in only one study [73]. Total TDP-43 quantified by SIMOA immunoassay in plasma-derived small EVs demonstrated very high diagnostic discrimination (area under the curve (AUC) of 0.99 of ALS cases against healthy controls, 0.85 of FTD against healthy controls, and 0.91 of ALS against FTD), with good consistency across two cohorts [24]. These AUCs were higher than for the TDP-43 in larger EVs (pelleted at 10,000 x*g*) at 0.92, 0.80 and 0.88, respectively. Small EV TDP-43 levels were also very low in a small number of FTD cases with confirmed *MAPT* mutations, and high in most cases with confirmed TDP-43 pathology, indicating potential as a biomarker to distinguish FTD cases by underlying tau or TDP-43 pathology. This work highlights the higher performance of small EVs, which contrasts with the findings of Casarotto et al. and Sproviero et al. where, in a small number of cases, the difference in TDP-43 levels between both ALS or FTD cases and healthy controls was highest in large EVs (pelleted at 20,000 x*g*) and not detected in small EVs [73, 77]. In the assessment by Casarotto et al. in sporadic FTD cases, immunoblotting demonstrated an increase in a 35 kDa TDP-43 fragment yet found no difference in full-length TDP-43. In ALS, using a total TDP-43 antibody, raised levels of a full-length doublet (presumed as phosphorylated and non-phosphorylated TDP-43) were observed [77]. These divergent findings between studies indicate the possibility that there may be specific forms of TDP-43 associated with EVs in different TDP-43 proteinopathies.

Only one study has examined the prognostic potential of EV-associated TDP-43 [24]. Here, plasma EV TDP-43 levels were associated with disease aggressiveness, and were significantly correlated with ALSFRS-R (functional) and ECAS (cognitive) scores (*r* = -0.40 and -0.53 respectively). Furthermore, plasma EV TDP-43 levels were more strongly associated with these clinical variables than were plasma neurofilament light levels (currently the leading prognostic biomarker for ALS), indicating that EV TDP-43 levels are not simply a reflection of the rate of axonal loss (*r* = -0.29 and -0.37 respectively).

Whilst these studies indicate great prognostic potential of TDP-43 levels in EVs, several questions are yet to be answered. Firstly, there has been little attempt to use single particle techniques to validate whether TDP-43 is directly associated with EVs in biofluids. Immuno-electron microscopy of plasma-derived EVs by Pasetto et al. indicated that there was no direct association of phosphorylated TDP-43 with vesicular structures, with labelling instead showing presence of co-isolated phosphorylated protein [34]. This is reminiscent of studies of alpha-synuclein in which a low proportion of the phosphorylated form was present inside EVs, raising questions about the biological relevance of the EV fraction [26, 27]. However, the ratio of phosphorylated (disease-associated form) to non-phosphorylated alpha-synuclein was 2-3-fold enriched in EVs compared to free protein, indicating that the EV membrane may be acting to aid the preservation and subsequent detection of the phosphorylated protein form, and therefore have valuable biomarker relevance [26]. Given that phosphorylated TDP-43 is believed to be most strongly associated with pathology, additional assessment of whether biofluid EVs carry this form is an essential question to answer.

Another issue is the high cross-reactivity of antibodies, which is especially problematic in biofluids, particularly when probing for very low abundance proteins like TDP-43. Indeed, evidence suggests that some TDP-43 antibodies show high cross-reactivity to albumin (55 kDa) and IgG (28 kDa and 50kDa) [34, 146]. Some reports of 25-28 kDa forms of TDP-43 in both cell culture and human samples have also been made using antibodies binding to the N-terminal region of the protein, highlighting the need for orthogonal validation approaches. Given the variability in sample matrix across different EV enrichment methods, careful validation of plate-based ELISA, SIMOA and NULISA assays, such as through spike-recovery to ensure linearity, is necessary. Comparison of total biofluid sample to matched EVs will also help to establish how specific disease-related differences are to EVs.

Immunocapture approaches hold the potential to improve separation of EVs from free protein and to enrich for rare populations, including separation of EVs by cell type of origin. L1CAM immunocapture in plasma-EVs has been applied in two patient cohorts: ALS/FTD [24] and LATE [46]. In the ALS/FTD cohort it was reported that TDP-43 in plasma EVs is almost exclusively found in L1CAM-positive EVs (both small and large), which is taken to imply a likely neuronal origin of the TDP-43. In the study of LATE, no differences in TDP-43 levels were seen in L1CAM-captured material using assays against either full-length or truncated (recognising residues 261–393) protein. However, when using SLC1A3 or TMEM119 for capture (as presumed astrocytic or microglial markers respectively), significant differences in TDP-43 levels were detected in LATE [46]. Given some evidence that pTDP-43 aggregates are highly abundant in peripheral tissues, such as skin, lymph node and gastrointestinal tract, additional validation of the specificity of these cell-type specific markers is greatly needed to identify whether these EVs are of *bona fide* central nervous system (CNS) origin [147].

In blood, the use of plasma versus serum, and the initial processing of these samples may also contribute to variation observed between studies. Coagulation involves the activation of platelets, releasing alpha-granules and dense granules alongside platelets-derived EVs, which constitute the most abundant cellular source of EVs in the blood [148]. Of the studies in Table 1 studying blood-derived EVs, one uses serum (i.e. fluid from coagulated blood) [145]two use plasma collected in sodium citrate anticoagulant tubes to inhibit platelet activation [73, 77] and use EDTA tubes [24, 34, 46, 47] although some subsequently add thrombin to thawed samples which induces platelet activation. Initial centrifugation speeds vary in these studies from 1000 x*g* to 3000 x*g*, with a second centrifugation step implemented in two studies [73, 77]. Temperature is not stated in the majority, but cold temperatures can again cause platelet activation and increase EV release [148]. Therefore, such variations in just initial processing steps will cause large differences in both the residual platelets contained within the plasma, and the release of additional platelet-derived EVs. Recent work has demonstrated that platelets are a major source of TDP-43 in plasma, with plasma from different sources showing a wide range in measured total TDP-43 [149]. Whilst there is not yet consensus on the optimal sample handling for either study of plasma-derived EVs, or for TDP-43 assays, these studies highlight the critical importance of detailed reporting to enable reproducibility. The International Society for Extracellular Vesicles has recently published MIBlood-EV guidelines to promote higher reporting standards across the EV field, which will aid in this regard [150].

## Extracellular vesicles as a source of other biomarkers in TDP-43 proteinopathies

### Altered EV secretion

Beyond a role as carriers of TDP-43, some evidence suggests that there may be alterations in EV secretion that reflect underlying cellular dysfunction and may be candidate biomarkers. *C9orf72* HRE may influence disease pathogenesis via both gain- and loss-of-function mechanisms [151–153]. Loss-of-function may be relevant in this context, as the C9orf72 protein functions as a regulator of Rab GTPases and plays an important role in the regulation of membrane trafficking processes in the endolysosomal system, which are intimately linked to EV biogenesis [154–158]. Reduced numbers of secreted particles have been reported in patient-derived fibroblasts and induced pluripotent stem cells derived motor neurons and astrocytes carrying *C9orf72* HRE [159, 160]. Some evidence suggests altered secretion pathways in other variants associated with TDP-43 proteinopathies. In mouse or patient-derived iPSC motor neurons carrying M337V TDP-43, fewer secreted particles were observed, but only under sodium arsenite-induced oxidative stress conditions [161]. In HeLa cells, however, siRNA-mediated knockdown of TDP-43 resulted in the increased secretion of LC3-II positive autophagic material present in EV fractions, whilst levels of canonical EV marker TSG101 were unchanged [76]. In patient-derived fibroblasts carrying *GRN* variants, secreted particle number was also reduced compared to healthy control lines, with a significant reduction also seen for CD63-positive particles [162]. *GRN* knockdown in HeLa cells, however, increased secreted levels of TSG101 levels alongside LC3-II, but with increased LC3-II only in mouse embryonic fibroblasts from *GRN* knockout mice, and no change in either protein in *GRN* knockdown mouse motor-neuron-like NSC-34 cells [76]. These studies raise the possibility that altered EV secretion might be a broader feature of TDP-43 proteinopathies. However, as these studies highlight, much remains to be elucidated, including whether this is restricted to specific cell types, and especially given reports of significant age- and sex -related differences in EV production [163–165]. Testing a broader panel of EV markers will aid in establishing an understanding of the alterations to secretion pathways in disease states.

There have also been some reports of differences in the number and size of EVs found in plasma [34, 77, 166–168]. However, in biofluids such as plasma these data are heavily influenced by the EV enrichment strategy, and largely rely on quantification by nanoparticle tracking analysis, which does not differentiate protein aggregates and lipoproteins from EVs [29, 169]. In a mouse TDP-43 Q331K model the number of particles enriched via nickel beads was significantly higher than in healthy controls, and with a smaller mean diameter, although this same difference was not observed in human plasma from participants with sporadic ALS [34]. In FTD, two studies using precipitation methods have reported a *decrease* in particle count in either *C9orf72* HRE or *GRN* variant carriers in plasma-derived EVs, whereas a study using an ultracentrifugation method has reported an *increase* in EVs for symptomatic *GRN* carriers [166–168]. Altered levels of high- and low-density lipoproteins in blood have separately been associated with risk of both ALS and FTD, so methods which enable the differentiation of the particle types are essential to the interpretation of these data [170–175]. Methods such as Raman spectroscopy, which may more effectively distinguish between particle types in a mixed population [176] or immunocapture methods, which are better able to physically separate EVs and lipoproteins, will aid further study in this area.

### Protein biomarkers in biofluid EVs

Shotgun proteomics of CSF has proved to be a powerful tool for biomarker discovery in ALS and FTD, identifying a range of proteins that are increased in abundance in ALS and show associations with disease prognosis. These are primarily proteins involved in inflammatory processes such as chitotriosidase-1 (CHIT1), chitinase-3-like protein-1 (CHI3L1 or YKL-40), chitinase-3-like protein-2 (CHI3L2 or YKL-39) and alpha 1-antichymotrypsin (SERPINA3) [142, 177–180]. However, CSF primarily contains classically secreted proteins and thus is potentially depleted of the intracellular signals directly linked to disease biology [181–183]. Highly abundant secreted proteins deriving from plasma (such as albumin and immunoglobulins) also mask detection of low abundance secreted proteins that derive from the CNS. Pre-purification of EVs offers one approach to overcoming these problems to enable discovery of circulating proteins that reflect the underlying cellular and tissue changes. In CSF, EVs were enriched for proteins of intracellular origin, indicating a greater potential to inform on cellular dysfunction in the brain and spinal cord than total CSF [33].

Cryptic mRNA and peptides for *STMN2*, *UNC13A* and *HDGFL2* have recently been demonstrated in brain tissue to be associated with brain regions containing high pTDP-43 in FTD, LATE and AD [184–187]. Some of these peptides are detectable in CSF and plasma, especially with the development of cryptic-specific antibodies, but current methods for detection lack sufficient sensitivity for accurate quantitation in biofluids [188, 189]. Pre-purification of EVs is therefore one method by which cryptic peptides might be enriched from whole biofluids to increase detectability and be used as an alternative readout of TDP-43 dysfunction.

Comparative proteomic profiling has been carried out on CSF-derived EVs from ALS patients and healthy controls [145, 190, 191]. In 3 ALS cases compared to 3 normal-pressure hydrocephalus controls, fourteen proteins differed in abundance (3 increased and 11 decreased) [190]. However, only Nucleolar complex protein 2 homolog (NOC2L; increased in abundance in ALS) was validated as likely directly associated with EVs in immuno-electron microscopy, whilst two others were found to be non-associated with EVs. NOC2L nuclear abundance was also decreased in the nuclei of motor neurons in the anterior horn of spinal cord in patients, reminiscent of the potential link between nuclear depletion and EV loading of TDP-43 protein. In the second study of 12 ALS cases (mixed sporadic and *C9orf72)*, compared to 4 healthy controls of individual proteins, only bleomycin hydrolase (BLMH) differed significantly after FDR correction, but at the pathway level there was a significant decrease in abundance of components of the proteasomal system [191]. Another study used an affinity-based proteomic approach to measure a panel of 92 proteins (primarily inflammation-associated) in total CSF and CSF EV preparations from ALS and controls [192]. No proteins were differentially abundant in EVs, whereas four proteins differed in total CSF. In the largest study of 20 ALS patients and 10 healthy controls, 246 proteins were significantly different in abundance [145]. Of these, 148 proteins were increased in ALS and were most highly enriched for “complement and coagulation cascades”. Ninety-eight proteins were decreased in ALS and were most highly enriched for “endoplasmic reticulum lumen”. These proteins showed high overlap with matched analysis from total serum-derived EVs, indicating a likely non-CNS source. An additional study of plasma-derived EVs similarly detected seven proteins which were increased in abundance in both discovery (12 ALS patients and 12 healthy controls) and validation (49 ALS patients and 20 healthy controls) cohorts; fibrinogen alpha, beta and gamma chains (FIBA, FIBB, FIBG), Von Willebrand factor (VWF), Lipopolysaccharide binding protein (LBP), Complement component C9 (C9) and proteoglycan-4 (PRG-4), again reflecting proteins non-CNS origin [193].

With advances in proteomics preparation and data acquisition, alterations in many of these individual proteins and pathways have been seen in total CSF without the need for pre-purification of EVs [142, 178, 194–196]. Many of these proteins are also known secreted proteins and may reflect a “corona” of positively charged proteins attracted to the negatively charged surface of EVs and may obscure characterisation of the “true” EV proteome [197]. Furthermore, these studies have used relatively large volumes of CSF (500 µL – 7.2 mL) compared to the volume required for proteomic analysis of total CSF, which is not feasible for large scale studies or clinical use. However, it is important to note that EV studies can be act as biomarker discovery studies to identify candidate proteins, which may be quantifiable using more sensitive immunoassay or targeted proteomic approaches for biomarker validation in whole CSF or blood. A further benefit of using EVs in CSF-based studies is that separation of EVs of different cell-type and subcellular origins is possible.

Several studies have investigated specific candidate proteins as biomarkers in EVs in ALS or FTD. For example, HSP90 was decreased in abundance in plasma-derived EVs from ALS and muscular dystrophy compared to healthy controls [34]. This decrease in HSP90 was recapitulated in plasma EVs from a TDP-43 Q331K mouse model [34]. Using the L1CAM immunocapture approach in plasma, Goeztl et al. also reported a reduction in the abundance of synaptic proteins synaptotagmin and synaptopodin in FTD (which was also seen in AD participants), although this study did not distinguish between TDP-43 and tau-related disease [198]. These candidate biomarkers have yet to undergo further validation in alternative patient cohorts.

### RNA biomarkers in biofluid EVs

In addition to protein cargo, EVs have been attributed as carriers of a wide variety of RNA subtypes including mRNA, snoRNA, tRNA and microRNA, which may change under different cellular conditions [21, 199]. Some RNA in EV preparations may be protected from degradation from RNAses, increasing its stability and thus the possibility of utilising it as a biomarker, for example for cryptic mRNAs in biofluids. Some studies have indicated the detection of full-length mRNAs within EVs [117, 200–202] although others have found an enrichment of mRNA fragments, which may limit use for detection of these cryptic mRNA species [199, 203–206]. Not all RNA in EV preparations, however, is sensitive to RNAse after detergent treatment, indicating that some fraction may be shielded from RNAse activity by other, non-vesicular protein complexes that may also hold biomarker potential [199, 207].

Non-targeted assessment of total RNA has been carried out by several studies using sequencing methods. Otake et al. carried out RNA sequencing of total (long and short) RNA in a small pilot study of CSF small EVs from four ALS patients and four healthy controls (1 mL per sample isolated by membrane affinity columns) [208]. Here, differentially abundant RNAs were enriched for gene ontology pathways including “unfolded protein response”, “protein ubiquitination pathway” and “response to oxidative stress”, with the differentially abundant mRNAs CUE domain-containing protein 2 (CUEDC2), Ras-related protein Rab-11 A (RAB11A), T-complex protein 1 subunit eta (CCT7) and Transmembrane protein 222 (TMEM222). In plasma, mRNA was also studied in both small and large EVs from 6 ALS and 9 FTD patients, alongside AD, PD and healthy control patients [209]. ALS and FTD samples together showed a reasonable degree of separation from other phenotypes in principal component analysis, with differential abundance in multiple splicing factors.

EV microRNAs have attracted more interest as potential biomarkers than mRNA. In FTD, screening of 752 microRNAs in polymer-precipitated EVs from CSF identified two miRNAs that were decreased in symptomatic versus pre-symptomatic states: miR-204-5p in *GRN*, *C9orf72* and sporadic FTD, and miR-632 in *GRN* and sporadic disease only [210]. Conversely, Tan et al. showed that the discrimination of FTD against healthy controls by miR-204-5p was poor with an AUC of 71.9%, with similar performance for AD [211]. Banack et al.. reported a panel of eight microRNAs in L1CAM-immunocaptured plasma EVs that differentiated ALS from healthy controls in multiple cohorts [212–215]. Another study assessed microRNAs in small and large plasma EVs in ALS and FTD cases compared to AD and PD, and identified a set of differential microRNAs that did not overlap with those identified in Banack et al. [216]. Other studies have suggested that microRNAs are extremely rare within EVs, including those derived from myoblasts and primary neurons [217–220]. Instead, they may be primarily associated with other extracellular, but non-vesicular, particles such as Argonaute complexes, low and high-density lipoproteins, and exomeres and supermeres, which are found abundantly in both plasma and CSF (Fig. 1) [221–225]. Although some potential microRNA biomarkers have been identified in EV fractions, it is possible that they are primarily localised to other co-isolating non-vesicular extracellular particles, so consideration of these alternative microRNA sources might improve both detectability and reproducibility.

## Conclusions

Many studies have investigated the role of EVs as carriers of TDP-43 with the primary focus on ALS and FTD. However, the recognition of TDP-43 pathology in LATE and AD further increases the potential clinical relevance of this work. There is currently a lack of consistency between studies, and at present it is not possible to distinguish what may be true disease-specific differences amongst TDP-43 proteinopathies, and what may be artefacts of the EV enrichment strategies employed. Characterising the specific particle populations under study, such as using proteomic and lipidomic approaches, will help to add clarity and comparability between studies. Emerging evidence indicates that the enrichment of EVs from blood can permit the detection of a form of TDP-43 discriminating TDP-43 and tau-related FTD, but much more work is needed to define (a) whether TDP-43 is directly associated with EVs – and thus which purification methods are most appropriate for reproducible detection, (b) the impact of pre-analytical variables such as plasma processing on reliable detection, (c) whether plasma TDP-43 is of CNS or peripheral origin, (d) which pathological form(s) this corresponds to (such as full-length, CTFs or phosphorylated forms), and (e) how these relate to known disease pathogenesis.

A significant question also remaining is whether interventions to increase the secretion of TDP-43 (such as apilimod which is currently in Phase 2 clinical trials), whether EV-associated or not, provide therapeutic benefit. In particular, it will be vital to carry out an assessment of the effects of such treatments in different genetic backgrounds, including *TARDBP*,* C9orf72* and *GRN.* This is of further importance given some limited evidence that EVs can contribute towards the spread of TDP-43 aggregation but much further investigation, especially in vivo, is needed. Understanding whether such EV-mediated transmission occurs by a direct templated seeding process, or indirectly via induction of cellular stress will also be necessary. Furthermore, there is a need to make use of in vitro and in vivo models to study the influence of different neuronal and glial cell types on the fate of extracellular TDP-43.

Beyond TDP-43, EVs offer a potential means to access CNS-relevant proteins and RNAs in peripheral biofluids, which may better reflect the intracellular dysfunction central to these diseases, for example in increasing sensitivity to detect cryptic products indicative of TDP-43 loss-of-function. In addition, immunocapture methods may enable differentiation of biomarker signatures by cellular and sub-cellular origins, providing further insights into the differing roles of neurons (and sub-types) and glia in the disease process. Whilst EV-based biomarker discovery may hold these benefits, time-consuming and often poorly reproducible methods of EV purification pose distinct challenges for their path to clinical utility. Therefore, careful validation of the specificity of these methodologies is an urgent necessity.

## Data Availability

No datasets were generated or analysed during the current study.

## Abbreviations

EV: Extracellular vesicle
CNS: Central nervous system
TDP-43: Trans-active response DNA binding protein 43 kDa
ALS: Amyotrophic lateral sclerosis
FTD: Frontotemporal dementia
MAPT: Microtubule-associated protein tau
LATE: Limbic predominant age-related TDP-43 encephalopathy
AD: Alzheimer’s disease
HRE: Hexanucleotide repeat expansion
CSF: Cerebrospinal fluid
SEC: Size exclusion chromatography
LDL: Low-density lipoprotein
VLDL: Very-low-density lipoprotein, HDL, high-density lipoprotein
CTF: C -terminal fragment
UC: Ultracentrifugation
TNT: Tunnelling nanotube
